# Attenuation of ferroptosis as a potential therapeutic target for neuropsychiatric manifestations of post-COVID syndrome

**DOI:** 10.3389/fnins.2023.1237153

**Published:** 2023-07-24

**Authors:** Ricardo A. L. Sousa, Asmaa Yehia, Osama A. Abulseoud

**Affiliations:** ^1^Department of Psychiatry and Psychology, Mayo Clinic Arizona, Phoenix, AZ, United States; ^2^Department of Medical Physiology, Faculty of Medicine, Mansoura University, Mansoura, Egypt; ^3^Department of Neuroscience, Graduate School of Biomedical Sciences, Mayo Clinic College of Medicine, Phoenix, AZ, United States

**Keywords:** ferroptosis, post-COVID syndrome, SARS-CoV-2, therapeutic target, long COVID

## Abstract

Coronavirus disease-19 (COVID-19), caused by severe acute respiratory syndrome coronavirus type 2 (SARS-CoV-2), is associated with the persistence of pre-existing or the emergence of new neurological and psychiatric manifestations as a part of a multi-system affection known collectively as “post-COVID syndrome.” Cognitive decline is the most prominent feature among these manifestations. The underlying neurobiological mechanisms remain under intense investigation. Ferroptosis is a form of cell death that results from the excessive accumulation of intracellular reactive iron, which mediates lipid peroxidation. The accumulation of lipid-based reactive oxygen species (ROS) and the impairment of glutathione peroxidase 4 (GPX4) activity trigger ferroptosis. The COVID-19-associated cytokine storm enhances the levels of circulating pro-inflammatory cytokines and causes immune-cell hyper-activation that is tightly linked to iron dysregulation. Severe COVID-19 presents with iron overload as one of the main features of its pathogenesis. Iron overload promotes a state of inflammation and immune dysfunction. This is well demonstrated by the strong association between COVID-19 severity and high levels of ferritin, which is a well-known inflammatory and iron overload biomarker. The dysregulation of iron, the high levels of lipid peroxidation biomarkers, and the inactivation of GPX4 in COVID-19 patients make a strong case for ferroptosis as a potential mechanism behind post-COVID neuropsychiatric deficits. Therefore, here we review the characteristics of iron and the attenuation of ferroptosis as a potential therapeutic target for neuropsychiatric post-COVID syndrome.

## Introduction

Ferroptosis is defined as a controlled form of cell death driven by excess intracellular labile iron and loss of the anti-oxidant enzyme glutathione peroxidase 4 (GPX4) activity, with consequent accumulation of lipid-based reactive oxygen species (ROS), especially lipid hydroperoxides (Yang and Stockwell, 2016). Interestingly, a knockout of GPX4 in mice led to lethality before embryonic day 9 (E9), which indicates a vital role for GPX4 in mouse development (Matsui, 1996; Yant et al., 2003). In the absence of GPX4, lipid peroxidation *in vivo* has a lethal nature, especially in neurons (Seiler et al., 2008). Alongside the unfavorable effect of GPX4 activity loss, iron overload contributes to the pathogenesis of coronavirus disease-19 (COVID-19), inciting inflammation, hypercoagulation, and immune dysfunction. Iron overload fosters an environment with free, unbound reactive iron, which triggers ROS generation (Habib et al., 2021). Ferritin, an iron storage protein, is a well-known inflammatory and iron overload biomarker, and is considered a direct mediator of the immune system in COVID-19 (Kappert et al., 2020; Kaushal et al., 2022; Lee et al., 2022). Iron overload is strongly suggested to contribute to the development of post-COVID neurological deficits (Fratta Pasini et al., 2021; Pandey et al., 2021; Zhang et al., 2022).

The post-COVID progressive and intense neurological clinical deterioration seems to occur due to the cytokine storm in COVID-19 patients (Para et al., 2022), which could create a vicious cycle with ferroptosis. Patients with COVID-19 presenting with high serum ferritin levels are usually in a severe condition (Abulseoud et al., 2022; Para et al., 2022). In addition, COVID-19 patients with comorbidities such as severe acute liver injury, diabetes, thrombotic complications, and cancer present with significantly higher levels of ferritin than those without (Cheng et al., 2020; Li et al., 2021).

A recent study analyzed COVID-19 effects over a 2-year retrospective cohort of 1,248,437 patients and revealed that cognitive decline, brain fog, and dementia are increasing over a 2-year follow-up period (Taquet et al., 2022). Another recent study by Wang et al. analyzed 6,245,282 older adults (over 65 years old), and the authors showed that older people infected by severe acute respiratory syndrome coronavirus 2 (SARS-CoV-2) were at significantly higher risk for a new diagnosis of Alzheimer’s disease (Wang et al., 2022). Post-COVID neurological consequences are tightly linked to other systems, including changes in the cardiovascular and immune systems, as well as higher levels of stress, anxiety, and depression (Campos et al., 2019; Salari et al., 2020; Zubair et al., 2020; Improta-Caria et al., 2021; Júnior et al., 2021; Sousa et al., 2021). The enigmatic nature of the post-COVID syndrome’s underlying mechanism necessitates intense investigation in order to achieve effective management strategy. Uncovering the role of iron as a potential therapeutic target is a critical step in pursuing better management of the post-COVID neurological consequences. Here, we review the characteristics of iron, and the attenuation of ferroptosis as a potential therapeutic target for neurological post-COVID syndrome.

## Iron chemistry

**Figure 1:**
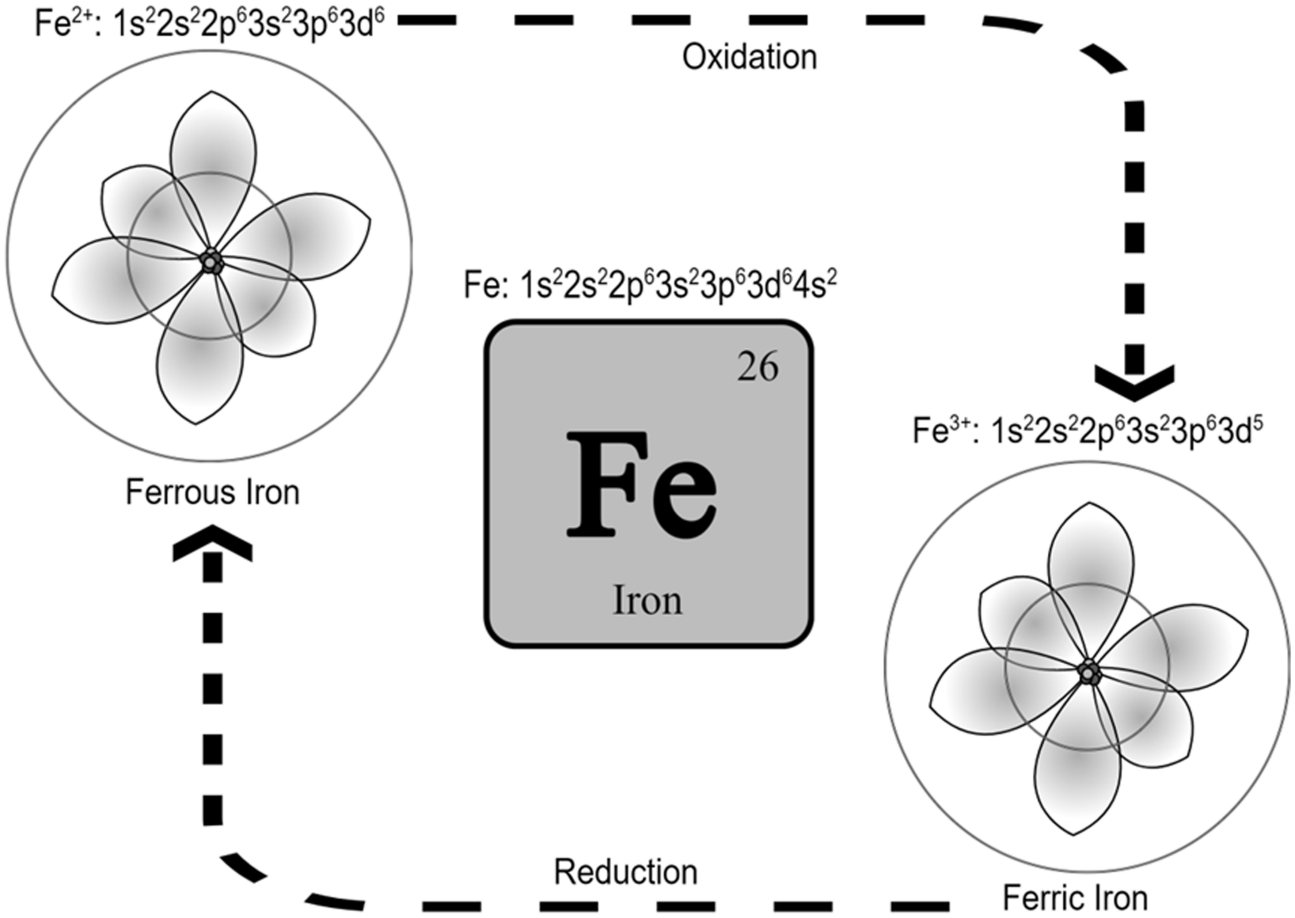
Iron and the two most common iron states in biological systems. It is called reduction when Fe^3+^ accepts an electron and becomes Fe^2+^, and it is called oxidation when Fe^2+^ donates an electron and becomes Fe^3+^. Schrodinger atom model is represented in the figures for ferrous and ferric iron.

Iron is the 26th element in the periodic table and is located in the transition metals group. It can exist in different oxidation states and possess catalytic properties (Neyens and Baeyens, 2003). Ferrous (Fe^+2^) and ferric (Fe^+3^) irons are the two most common iron states in biological systems (Cabantchik, 2014). From an atomic orbital energy standpoint, a ferrous iron atom has a total of 26 electrons distributed in the following manner: two electrons in 1 s (the least energy orbit), 2 electrons in 2 s, 6 electrons in 2p, 2 electrons in 3 s, 6 electrons in 3p, 2 electrons in 4 s, and 6 electrons in the 4d orbit (Fe: 1s^2^2s^2^2p^6^3s^2^3p^6^3d^6^4s^2^). The position energy in the 3^rd^ orbit (3d) is slightly higher than the position energy in the 4^th^ orbit (4 s). This means that electrons will fill the 4 s position first before filling the 3d position, and also that the 4 s electrons will be lost first before the 3d electrons (Sherry and Fürstner, 2008). That is why ferrous iron [(Fe^2+^): 1s^2^2s^2^2p^6^3s^2^3p^6^3d^6^] has lost two electrons from the 4 s position, while ferric iron [(Fe^3+^): 1s^2^2s^2^2p^6^3s^2^3p^6^3d^5^] has lost a total of three electrons (two from the 4 s and one from the 3d position). As such, ferric iron (Fe^3+^) is relatively more stable than ferrous iron (Fe^2+^). Stability means the balance between positive and negative charges in the atom. Changing the number of electrons disturbs this balance. The atom holds electrons through electron binding energy, which is the minimum energy required to remove an electron from an atom. This energy is directly proportional to the atomic number (heavier atoms have more energy) and inversely proportional to the distance from the nucleus (electrons in outer orbits require less energy to be removed from the atom). As stated, ferrous iron has lost two electrons from 4 s, leaving six electrons in 3d, while ferric iron has lost two electrons from 4 s and one from 3d leaving five electrons in 3d. Electrons in partially filled orbits require more energy to remove from the orbit compared to electrons in fully filled orbits, which is why ferric iron is more stable compared to ferrous iron. The ability of ferric iron (Fe^3+^) to accept an electron and become ferrous iron (Fe^2+^) is called reduction, and the ability of ferrous iron (Fe^2+^) to donate an electron and become ferric iron (Fe^3+^) is called oxidation (Figure 1).

This reduction–oxidation or Redox, is what makes iron a catalyst for reactions that require electron transfer (Hosseinzadeh and Lu, 2016). In the Fenton reaction, for example, iron catalyzes the decomposition of hydrogen peroxide (H_2_O_2_) to produce hydroxyl radicals (^●^OH) at an acidic PH with high oxidizing properties (Neyens and Baeyens, 2003) [see the reaction below. The unpaired electron of a free radical is represented with a dot (^●^)].


$$H_{2}O_{2}+{\text{Fe}}^{3+}\to {\text{Fe}}^{2+}+H^{+}+{\text{HO}}^{•}{\;}_{2}$$

$$H_{2}O_{2}+{\text{Fe}}^{2+}\to {\text{Fe}}^{3+}+{\text{OH}}^{-}+{\;}^{•}\text{OH}$$

Numerous key enzymes rely on iron redox properties such as the mitochondrial respiratory chain enzymes (Gille and Reichmann, 2011), the aconitase enzyme in the tri-carboxylic acid cycle, which facilitates the conversion of citrate to iso-citrate via cis-aconitate (Kennedy et al., 1983; Robbins and Stout, 1989), and tyrosine hydroxylase, which is the rate-limiting enzyme in catecholamine synthesis (Ramsey et al., 1996; Daubner et al., 2011). Moreover, the serotonergic system may require ferrous iron (Fe^2+^) for binding serotonin to serotonin-binding proteins (Tamir and Liu, 1982). As such, iron is beneficial in regulating energy production and neurotransmitter synthesis, such as glutamate (McGahan et al., 2005; Lall et al., 2008) and dopamine (Ramsey et al., 1996). However, the same inherent “pro-oxidant” ability of ferrous iron (Fe^2+^) to donate an electron and catalyze enzymatic reactions also causes hydrogen peroxide (H_2_O_2_) to breakdown into hydroxyl radicals (^●^OH) causing lipid peroxidation and oxidative damage to proteins, deoxyribonucleic acid (DNA), and ribonucleic acid (RNA) (Graham et al., 2007). For this reason, the oxidative property of ferrous iron (Fe^2+^) must be managed by transforming it into a more stable ferric iron (Fe^3+^) and shielding this ferric iron (Fe^2+^) from water by transporting ferric iron into protein transporters.

The liver is the major organ involved in iron homeostasis. The liver produces iron transporters, including transferrin and divalent metal transporter-1 (DMT1). Transferrin transports ferric iron in the intestinal lumen and plasma, while DMT1 transports ferrous iron from the interior of the endosome to the cytosol. The liver also produces ferritin and hepcidin, which serve as the iron storage protein, and the only iron hormonal regulator, respectively. Furthermore, it produces transferrin receptor 1 (TfR1) and the iron responsive element binding proteins, which act as iron sensors and regulate the mRNA iron responsive element to enhance transferrin receptor translation and to down-regulate ferritin translation (Winterbourn, 1995). Moreover, the liver controls the oxidative state of iron by producing ferrireductase and ferroxidase enzymes. Ferrireductase reduces endosomal ferric iron, at low PH, within endocytosis vesicles into ferrous iron before it is released from the transferrin and reduces stored ferric iron into ferrous iron before release from ferritin, while ferroxidase oxidizes ferrous iron into ferric iron to be stored in ferritin (Graham et al., 2007).

## Iron absorption and fate within the cell

Dietary ferric iron uptake by the intestinal mucosa depends on hepatic transferrin, Transferrin Receptor 1 (TfR1), and iron content within enterocytes. Transferrin is synthesized by hepatocytes and excreted through the biliary system into the intestinal lumen. Transferrin binds two atoms of iron per protein molecule and brings them into the cells by endocytosis (Bezkorovainy, 1989b). Duodenal and upper jejunal (low PH), but not ileal (Huebers et al., 1983), enterocyte crypts express TfR1 at the basolateral border (Barisani and Conte, 2002). These cells uptake iron from plasma transferrin by receptor-mediated endocytosis (Morgan and Oates, 2002). TfR1 is involved in sensing body iron stores. TfR1 expression increases with iron deficiency and decreases with iron overload (Barisani and Conte, 2002). Within endosomes, transferrin, TfR1, and ferric iron (Fe^3+^) are subject to low PH to separate ferric iron (Fe^3+^) from transferrin and TfR1. Transferrin and TfR1 are cycled back to the cell surface or plasma, while ferric iron (Fe^3+^) is reduced by the ferrireductase enzyme into ferrous iron (Fe^2+^). DMT1 transports ferrous iron (Fe^2+^) out of the endosome and into the cytoplasm, where it enters a transient pool of metabolically active iron known as the labile iron pool (LIP). LIP iron can be utilized for cellular processes such as DNA synthesis, repair, and cell cycling. Alternatively, excess LIP iron can be stored in ferritin (Paul et al., 2017) or exit the cell through ferroportin. Iron storage as ferric iron (Fe^3+^) within ferritin protein may occur (Ponka et al., 1998; Zandman-Goddard and Shoenfeld, 2007; Finazzi and Arosio, 2014). However, iron must be in the ferrous state (Fe^2+^) to enter and exit the ferritin molecule (Crichton, 1973; Bezkorovainy, 1989a; Hintze and Theil, 2006). The enzymes ferroxidase and ferrireductase change the state of iron back and forth between ferric (Fe^3+^) and ferrous (Fe^2+^). Ferritin synthesis is up regulated by several factors, including high toxic oxygen radical or cytokine concentrations, typically seen during infections. High ferritin production reduces the bioavailability of iron which leads to less reactive oxygen radical production (Koorts and Viljoen, 2007; Zandman-Goddard and Shoenfeld, 2007).

On the other hand, rapid degradation of ferritin could be toxic due to the uncontrolled release of free reactive iron. However, degradation within membrane-encapsulated “secondary lysosomes’ may avoid this problem through the formation of hemosiderin, which is another form of iron storage protein (Harrison and Arosio, 1996). Iron exit depends on the iron export channel ferroportin expression and the hepatic hormone hepcidin concentration. Ferroportin is an iron transporter on the surface of absorptive enterocytes, hepatocytes, and other cells. The main function of ferroportin is to export ferrous iron from iron-containing cells into plasma transferrin as ferric iron (Fe^3+^). The ferroxidase enzyme oxidizes ferrous iron into ferric iron (Nemeth et al., 2004; Ganz, 2005, 2006, 2007; Drakesmith et al., 2015). Hepcidin is an iron-regulatory hormone synthesized in hepatocytes. Hepcidin binds, internalizes, and degrades the cellular iron exporter ferroportin and thereby decreases iron efflux into plasma. Hepcidin synthesis is stimulated by high plasma iron and iron stores and inhibited by erythropoietic activity (Ganz, 2007). Hepcidin deficiency causes iron overload in hereditary hemochromatosis and ineffective erythropoiesis (Ganz and Nemeth, 2012; Ginzburg, 2019).

## Brain iron uptake

Blood transferrin binds to transferrin receptors on epithelial cells of the choroid plexus and oligodendrocytes. Among glial cells, oligodendrocytes synthesize 90% of brain transferrin since iron plays a significant role in their development and in myelin formation (Todorich et al., 2009). Neurons and glial cells take up iron released into the brain interstitium, and apo-transferrin is recycled back to the blood (Bloch et al., 1987; Crowe and Morgan, 1992; Moos, 2002). This process increases during the period of rapid brain growth and iron deficiency and declines with age (Taylor and Morgan, 1990). It also can be reversed (from the brain interstitium back to the blood) during brain iron overload (Broadwell, 1989). Most iron entering the brain across the capillary endothelium finally leaves the system with the bulk outflow of the cerebrospinal fluid (CSF) through the arachnoid villi and other channels (Bradbury, 1997). Interestingly, approximately half of the transferrin in the CSF is derived from the choroid plexus, while the other half comes from the blood in the adult brain (Crowe and Morgan, 1992). Neuronal function is iron-dependent because of the high energy demand, oxidative metabolism, and cytochrome participation in the respiratory chain. The function of oligodendrocytes is also iron-dependent since iron is involved in lipid synthesis needed for myelin synthesis (Connor and Menzies, 1996). Microglial iron is essential for the inflammatory release of hydrolytic enzymes and free radicals via the oxidation of ferrous iron. Microglial iron also participates in the formation of nitric oxide, where iron acts as a co-factor for the nitric oxide synthase enzyme that catalyzes the formation of nitric oxide from the amino acid L-arginine (Moos, 2002). Several iron-related molecular pathways have been reported to be involved in COVID-19 (Farahani et al., 2022).

## Iron-related molecular mechanisms in COVID-19

Uncovering the molecular mechanisms involved in SARS-CoV-2 infection is crucial for a better understanding and management of COVID-19’s consequences. The angiotensin converting enzyme 2 (ACE2)/Angiotensin 1–7 Mas receptor pathway is an important part of the renin-angiotensin system (RAS), which converts angiotensin II into a heptapeptide (Angiotensin 1–7) and angiotensin I into a nonapeptide (Angiotensin 1–9). ACE2 works as a cell surface receptor through which SARS-CoV-2 can enter the cell (De Sousa et al., 2021; Farahani et al., 2022). Viral brain invasion occurs through the olfactory nerve, infection of the vascular endothelium, or migrating leukocytes crossing the blood–brain barrier (Zubair et al., 2020). SARS-CoV-2 infection leads to higher levels of ROS that will cause harmful effects on proteins, lipids, and DNA, creating a similar state to cell necrosis (Kouhpayeh et al., 2021). Ferroptosis is considered a novel type of cell death that shares some aspects with cell necrosis (Anthonymuthu et al., 2021). The excess of iron in the plasma and body organs is tightly related to COVID-19 (Liu P. et al., 2020; Habib et al., 2021; Li et al., 2021; Zhang et al., 2022).

Among the COVID-19 related molecular pathways, there are 22 pathways identified (RAS, NF-kappa B, mTOR, Notch, HIF-1, MAPK, JAK–STAT, TNF signaling pathway, autophagy, apoptosis, necroptosis, B cell receptor signaling pathway, chemokine signaling pathway, IL-17 signaling pathway, natural killer cell mediated cytotoxicity, NOD-like receptor signaling pathway, T cell receptor signaling pathway, Th1 and Th2 cell differentiation, Th17 cell differentiation, toll-like receptor signaling pathway, complement and coagulation cascades, and cytokine-cytokine receptor interaction pathway) with non-cross-talk genes and cross-talk genes making up 561 genes. The cytokine-cytokine receptor interaction pathway is the most significant pathway, presenting 197 crosstalk genes of the 561 total genes (Farahani et al., 2022). ADAM17 is also identified as an important mediator of the major signaling pathways involved in the deleterious consequences of COVID-19 since it processes various substrates, like membrane-anchored cytokines, growth factors, cell adhesion molecules, receptors, and other proteins. The reported damage to body organs and brain regions in COVID-19 results mainly from the cytokine storm, one of the main SARS-CoV-2 infection harmful consequences (Li et al., 2020).

**Figure 2:**
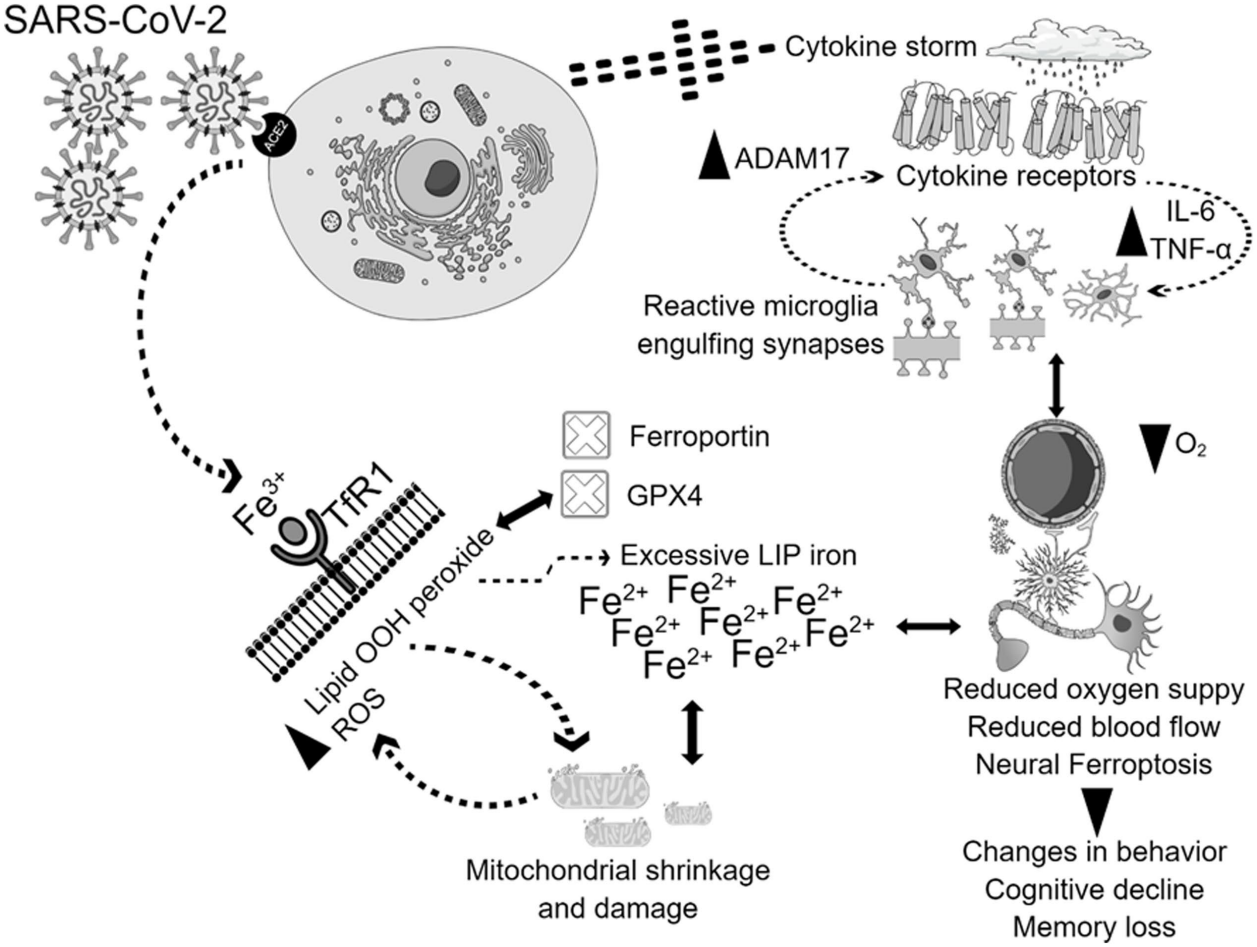
Potential mechanisms of physiological and neurological ferroptosis-associated changes due to SARS-CoV-2 infection. SARS-CoV-2 virus enters the cells through the ACE2 receptor, and triggers cytokine storm, and ferroptosis, which together contributes to mitochondrial damage, and microglial activation. The activation of all these mechanisms together contribute to the enhancement of ADAM17, TNF-, IL-6, with simultaneous inhibition of GPX4, ferroportin, reduction of oxygen supply, and blood flow. These physiological and neurological changes will lead to behavioral disturbances, cognitive decline, and memory loss.

Greater levels of inflammatory cytokines combined with a hypoxic state resulting from pulmonary dysfunction can lead to a reduction in blood flow and oxygen supply (Fratta Pasini et al., 2021). The cytokine storm is a prominent feature of the SARS-CoV-2 infection, instigating systemic flooding with pro-inflammatory cytokines such as interleukin-6 (IL-6), IL-1β, IL-8, interferon-γ (IFN-γ), tumor necrosis factor-alpha (TNF-α), monocyte chemo-attractant protein-1 (MCP-1), and macrophage inflammatory protein-1A (MIP-1A) (Fara et al., 2020; Kempuraj et al., 2020). Moreover, the cytokine storm co-exists with a massive increase in coagulopathies and acute phase reactants such as C-reactive protein (CRP) and serum ferritin which correlate with the severity of the disease (Cheng et al., 2020; Lino et al., 2021; Savla et al., 2021). High levels of peripheral pro-inflammatory cytokines compromise the blood brain barrier (BBB) integrity, cross over to the brain vicinity, and activate its resident immune cells, causing microglial activation which in turn creates a medium of neuroinflammation (Almutairi et al., 2021). Interleukin-6 (IL-6) stimulates the synthesis of ferritin and hepcidin in a cytokine storm (Daher et al., 2017; Bessman et al., 2020). Hepcidin and hepcidin-like proteins bind to ferroportin, the cellular iron exporter, which prevents iron outflow and contributes to enhanced LIP, posing the risk of the Fenton reaction and ferroptosis when GPX4 does not eliminate the excess lipid ROS (Frazer and Anderson, 2014; Ganz, 2018). Hoarding iron into the cell as in cases of iron overload could be detrimental since SARS-CoV-2 replication requires iron (Liu W. et al., 2020). Furthermore, SARS-CoV-2 attacks hemoglobin, leading to iron release into the circulation (Zhang et al., 2022). Therefore, the interaction between the cytokine storm and iron dysregulation, with potential subsequent ferroptosis in COVID-19, could activate molecular mechanisms that result in brain damage. In that case, brain damage could heavily rely on higher hepcidin levels, excessive iron influx through transferrin receptors, and the release of free iron into the circulation due to infection. In addition, during ferroptosis, mitochondria stop elongating, condense, and reduce in size and number. Microglia get activated, engulf synapses, and are polarized to a pro-inflammatory phenotype, flooding the brain with pro-inflammatory cytokines such as tumor necrosis factor alpha (TNF-α), leading to changes in cognition and behavior (Figure 2; Zhang et al., 2022).

## Ferroptosis inhibition: a possible therapeutic target for neurological post-COVID syndrome

Ferroptosis can be classified as a new type of cell death that is dependent on lipid peroxidation and characterized by mitochondrial shrinkage (Liu P. et al., 2020). Ferroptosis contributes to the development of several pathologic processes, including inflammation and neurodegenerative diseases (Zhang et al., 2022). Multiple neurodegenerative diseases present with iron accumulation and lipid peroxidation in the brain (Farahani et al., 2022). Hambright et al. tested the efficacy of tamoxifen-induced deletion of forebrain neuronal GPx4 gene (Gpx4BIKO mice) as a model of ferroptosis. Gpx4BIKO mice exhibited significant deficits in spatial learning and memory function associated with lipid peroxidation and hippocampal neurodegeneration. Treatment with the ferroptosis inhibitor liproxstatin-1 ameliorated neurodegeneration (Hambright et al., 2017). Recent studies have also documented the role of ferroptosis in mediating cognitive dysfunction in animal models of Alzheimer’s disease (AZ). Using the 5xFAD mouse model (has low GPx4 and cognitive impairment), Chen et al. generated a 5xFAD mice that overexpress Gpx4 (5xFAD/GPX4). These mice with overexpressed GPx4 performed significantly better in memory and learning tasks compared to the control 5xFAD mice and had reduced neurodegeneration (Chen et al., 2022). Bao et al. showed that selective genetic deletion of ferroportin 1 led to ferroptosis, hippocampal atrophy and memory deficits, while restoring ferroportin 1 ameliorated ferroptosis and memory impairment in the APPswe/PS1dE9 mouse model of AZ (Bao et al., 2021). Along the same lines, Hao et al. showed that cognitive dysfunction in the streptozotocin rat model of type 1 diabetes is related to hippocampal iron overload and ferroptosis mediated by down regulation of ferroportin 1 gene (Hao et al., 2021). A growing body of evidence suggests ferroptosis as a plausible mechanism behind the SARS-CoV-2-associated neuropsychiatric symptoms, cognitive decline, and memory loss (Zhang et al., 2022). In an ischemic stroke model, Acyl-coenzyme A synthase long-chain family member 4 (ACSL4), which is crucial to ferroptosis-related lipid peroxidation, promoted ferroptosis-induced brain injury and neuroinflammation with similar findings to neuro-COVID-19 events, such as infarct size increase, reduced neurological function, microglial activation, and increased pro-inflammatory cytokines (Cui et al., 2021). In the context of COVID 19, we can see glimpses of potential iron perturbation along with neuroinflammation in several forms. High serum ferritin (Cheng et al., 2020) and hepcidin levels (Hortová-Kohoutková et al., 2023), low serum iron levels (Gaiatto et al., 2023), and low transferrin saturation (Claise et al., 2022) have been significantly correlated with COVID-19 severity, hospitalization, and mortality (Zhou et al., 2020; Kaushal et al., 2022; Suriawinata and Mehta, 2022). In COVID-19 patients, altered iron metabolism, depletion of glutathione (GSH) (Kumar et al., 2022), inactivation of GPX4 (Muhammad et al., 2021), and up regulation of lipid peroxidation biomarkers strongly propose ferroptosis as a plausible mechanism for COVID-19 multi-organ affection, including neuropsychiatric sequelae (Yang and Lai, 2020).

It is repeatedly reported that the use of multiple iron chelators such as deferoxamine prevents the formation of ROS by averting electron donation from iron to oxygen, which could minimize ferroptosis (Ren et al., 2020; Anthonymuthu et al., 2021). To prevent ferroptosis, ferrostatin-1 plays a role as a lipid ROS scavenger (Yang and Stockwell, 2016). Ferrostatin-1 alleviates angiotensin II-induced inflammation and ferroptosis by inhibiting the enhancement of ROS levels in astrocytes and the subsequent reactive gliosis (Li et al., 2021). Another recent study showed that ferrostatin-1 diminishes the levels of ROS and malondialdehyde and enhances superoxide dismutase activity in HT-22 cells, revealing a protective effect of this ferroptosis inhibitor (Chu et al., 2020). Curiously, a recent study reported that ferrostatin-1, in the presence of reduced iron levels, eliminates lipid hydroperoxides, presenting a similar effect as GPX4 (Miotto et al., 2020). The use of deferoxamine as a ferroptosis inhibitor showed success in reducing inflammation and improving memory in different models of neurodegenerative diseases (Xue et al., 2016; Fine et al., 2020; Lee et al., 2021). These results propose ferroptosis inhibition as a plausible approach to managing the post-COVID neurological disturbances (Table 1).

**Table 1:** Ferroptosis inhibitors/ROS scavengers.

Ferroptosis inhibitors/ROS scavengers	Animal model	Effect	Reference
Liproxtatin-1	Tamoxifen-induced deletion of GPx4 gene (Gpx4BIKO mice), model of ferroptosis	Ameliorated spatial learning and memory function along with lipid peroxidation and hippocampal neurodegeneration.	Hambright et al. (2017)
Liproxtatin-1	Endovascular perforation model of sub arachnoid hemorrhage in Male C57BL/6 mice.	Attenuated the neurological deficits and brain edema, reduced neuronal cell death, restored the redox equilibrium, and preserved GPX4. It also decreased the activation of microglia and the release of IL-6, IL-1β, and TNF-α.	Cao et al. (2021)
Liproxtatin-1	LPS-Induced Cognitive Impairment in male C57BL/6 mice.	Ameliorated memory impairment induced by LPS. It decreased the microglial activation and the production of IL-6 and TNF-α, attenuated oxidative stress and lipid peroxidation, and alleviated mitochondrial injury and neuronal damage after LPS exposure. It decreased iron deposition and regulated the ferroptosis-related proteins; transferrin, heavy ferritin, mitochondrial ferritin and Gpx4.	Li et al. (2022)
Liproxtatin-1	Complete Freund’s adjuvant (CFA)-induced inflammatory pain in male adult Sprague–Dawley (SD) rats	Intrathecal liproxstatin-1 improved mechanical and thermal hypersensitivities in CFA rats. It inhibited ferroptosis in the spinal cord and dorsal root ganglion tissues of CFA rats. It alleviated lipid peroxidation, disorders of anti-acyl-coenzyme A synthetase long-chain family member 4 (ACSL4) and GPX4.	Deng et al. (2023)
Liproxtatin-1	Type 2 diabetes (T2D)-associated cognitive dysfunction in HFD-fed C57BL/6 mice injected with low-dose streptozotocin.	Attenuated iron accumulation and oxidative stress response, resulting in better cognitive function.	Xie et al. (2023)
Ferrostatin-1	Kainic-acid model of temporal lobe epilepsy in adult male Sprague–Dawley rats	It improved cognitive functions in epileptic rats by inhibiting P38 MAPK and in turn increasing the expression of synaptophysin (SYP) and postsynaptic density protein 95 (PSD-95) in the hippocampus.	Ye et al. (2020)
Ferrostatin-1	Angiotensin II-induced inflammation in mouse primary cortical astrocytes isolated from CD-1 mice.	It suppressed the Ag II-induced increase of angiotensin 1 receptors, IL-6, IL-1β, and GFAP in the astrocytes. It upregulated the decreased GPx4, GSH, Nrf2, and HO-1 in the astrocytes induced by Ang II, denoting decreased inflammation and ROS production.	Li et al. (2021)
Ferrostatin-1	Amyloid beta (25–35)-injected Wistar rats model of Alzheimer’s disease	It reversed the Aβ-induced spatial learning and memory impairment and enhanced the neuropathological changes such as better cell survival and less intracellular Aβ deposits. Levels of GPX4 and SLC7A11 were improved.	Naderi et al. (2023)
Ferrostatin-1	Middle cerebral artery occlusion (MCAO) model of cerebral ischemia/reperfusion injury in male C57BL/6 mice	It reduced high iron levels demonstrated in the stroke model. It also decreased lipid peroxidation with lower levels of malondialdehyde. It increased the levels of GSH and the expression of SLC7A11 and GPX 4. It reduced the infarct size and improved the neurobehavioral outcomes.	Liu et al. (2023)
Ferrostatin-1	Bupivacaine (BUP)-Induced spinal neurotoxicity in Sprague–Dawley male rats	Intrathecal ferrostatin-1 improved rats functional recovery, histopathological outcomes, and neural survival. It reversed the e BUP-induced ferroptosis-related mitochondrial shrinkage. It decreased lipid peroxidation products such as malondialdehyde (MDA) and 4-hydroxynonenal (4HNE). It inhibited the ROS accumulation and restored normal levels of GPX4, GSH, and SLC7A11.	Zhao et al. (2023)
GPX4	5xFAD Alzheimer’s mouse model	Mice with overexpressed GPx4 performed significantly better in memory and learning tasks and had reduced neurodegeneration	Chen et al. (2022)

## Conclusion

The attenuation of ferroptosis as a potential therapeutic target for neurological post-COVID syndrome is not yet fully established. However, inactivation of GPX4 and up regulation of lipid peroxidation and ROS are constitutive components of both SARS-CoV-2 infection and ferroptosis, suggesting a potentially major role for ferroptosis inhibitors. Identifying the possible beneficial molecular changes in the brain caused by these inhibitors in the context of COVID-19 would provide a great insight into managing post-COVID neuropsychiatric manifestations.

## Author contributions

OA: concept and design, drafting of the manuscript, and supervision. All authors: critical revision of the manuscript for important intellectual content.

## Funding

This work was funded by the Department of Psychiatry and Psychology at the Mayo Clinic Arizona.

## Conflict of interest

The authors declare that the research was conducted in the absence of any commercial or financial relationships that could be construed as a potential conflict of interest.

## Publisher’s note

All claims expressed in this article are solely those of the authors and do not necessarily represent those of their affiliated organizations, or those of the publisher, the editors and the reviewers. Any product that may be evaluated in this article, or claim that may be made by its manufacturer, is not guaranteed or endorsed by the publisher.

## References

[ref1] Abulseoud O. A. Yehia A. Egol C. J. Nettey V. N. Aly M. Qu Y.. (2022). Attenuated initial serum ferritin concentration in critically ill coronavirus disease 2019 geriatric patients with comorbid psychiatric conditions. Front. Psych. 13:1035986. doi: 10.3389/fpsyt.2022.1035986, PMID: 36440432 PMC9681793

[ref2] Almutairi M. M. Sivandzade F. Albekairi T. H. Alqahtani F. Cucullo L. (2021). Neuroinflammation and its impact on the pathogenesis of COVID-19. Front. Med. 8:745789. doi: 10.3389/fmed.2021.745789, PMID: 34901061 PMC8652056

[ref3] Anthonymuthu T. S. Tyurina Y. Y. Sun W. Y. Mikulska-Ruminska K. Shrivastava I. H. Tyurin V. A.. (2021). Resolving the paradox of ferroptotic cell death: Ferrostatin-1 binds to 15LOX/PEBP1 complex, suppresses generation of peroxidized ETE-PE, and protects against ferroptosis. Redox Biol. 38:101744. doi: 10.1016/j.redox.2020.101744, PMID: 33126055 PMC7596334

[ref4] Bao W.-D. Pang P. Zhou X.-T. Fan H. Xiong W. Chen K.. (2021). Loss of ferroportin induces memory impairment by promoting ferroptosis in Alzheimer’s disease. Cell Death Different. 28, 1548–1562. doi: 10.1038/s41418-020-00685-9 PMC8166828 33398092

[ref5] Barisani D. Conte D. (2002). Transferrin receptor 1 (TfR1) and putative stimulator of Fe transport (SFT) expression in iron deficiency and overload: an overview. Blood Cells Mol. Dis. 29, 498–505. doi: 10.1006/bcmd.2002.0588, PMID: 12547240

[ref6] Bessman N. J. Mathieu J. R. R. Renassia C. Zhou L. Fung T. C. Fernandez K. C.. (2020). Dendritic cell-derived hepcidin sequesters iron from the microbiota to promote mucosal healing. Science 368, 186–189. doi: 10.1126/science.aau6481, PMID: 32273468 PMC7724573

[ref7] Bezkorovainy A. (1989a). Biochemistry of nonheme iron in man. I. Iron proteins and cellular iron metabolism. Clin. Physiol. Biochem. 7, 1–17. PMID: 2665999

[ref8] Bezkorovainy A. (1989b). Biochemistry of nonheme iron in man. II. Absorption of iron. Clin. Physiol. Biochem. 7, 53–69. PMID: 2667838

[ref9] Bloch B. Popovici T. Chouham S. Levin M. J. Tuil D. Kahn A. (1987). Transferrin gene expression in choroid plexus of the adult rat brain. Brain Res. Bull. 18, 573–576. doi: 10.1016/0361-9230(87)90122-5, PMID: 3300864

[ref10] Bradbury M. W. (1997). Transport of iron in the blood-brain-cerebrospinal fluid system. J. Neurochem. 69, 443–454. PMID: 9231702 10.1046/j.1471-4159.1997.69020443.x

[ref11] Broadwell R. D. (1989). Transcytosis of macromolecules through the blood-brain barrier: a cell biological perspective and critical appraisal. Acta Neuropathol. 79, 117–128. doi: 10.1007/BF00294368, PMID: 2688350

[ref12] Cabantchik Z. I. (2014). Labile iron in cells and body fluids: physiology, pathology, and pharmacology. Front. Pharmacol. 5:45. doi: 10.3389/fphar.2014.00045 24659969 PMC3952030

[ref13] Campos B. P. e. S. dos Santos Gomes G. D. de Sousa Braz A. de Vilela A. T. (2019). Cardiovascular risk factors and risk measurement in patients with psoriatic arthritis in a university hospital. Int. J. Cardiovasc. Sci. 33, 112–118.

[ref14] Cao Y. Li Y. He C. Yan F. Li J.-R. Hang-Zhe X.. (2021). Selective ferroptosis inhibitor liproxstatin-1 attenuates neurological deficits and neuroinflammation after subarachnoid hemorrhage. Neurosci. Bull. 37, 535–549. doi: 10.1007/s12264-020-00620-5, PMID: 33421025 PMC8055759

[ref15] Chen L. Dar N. J. Na R. McLane K. D. Yoo K. Han X.. (2022). Enhanced defense against ferroptosis ameliorates cognitive impairment and reduces neurodegeneration in 5xFAD mice. Free Radic. Biol. Med. 180, 1–12. doi: 10.1016/j.freeradbiomed.2022.01.002, PMID: 34998934 PMC8840972

[ref16] Cheng L. Li H. Li L. Liu C. Yan S. Chen H.. (2020). Ferritin in the coronavirus disease 2019 (COVID-19): a systematic review and meta-analysis. J. Clin. Lab. Anal. 34:e23618. doi: 10.1002/jcla.23618 33078400 PMC7595919

[ref17] Chu J. Liu C. X. Song R. Li Q. L. (2020). Ferrostatin-1 protects HT-22 cells from oxidative toxicity. Neural Regen. Res. 15, 528–536. doi: 10.4103/1673-5374.266060, PMID: 31571665 PMC6921338

[ref18] Claise C. Saleh J. Rezek M. Vaulont S. Peyssonnaux C. Edeas M. (2022). Low transferrin levels predict heightened inflammation in patients with COVID-19: new insights. Int. J. Infect. Dis. 116, 74–79. doi: 10.1016/j.ijid.2021.12.340, PMID: 34952211 PMC8688186

[ref19] Connor J. R. Menzies S. L. (1996). Relationship of iron to oligodendrocytes and myelination. Glia 17, 83–93. doi: 10.1002/(SICI)1098-1136(199606)17:2<83::AID-GLIA1>3.0.CO;2-7, PMID: 8776576

[ref20] Crichton R. R. (1973). Structure and function of ferritin. Angew. Chem. Int. Ed. Engl. 12, 57–65. doi: 10.1002/anie.197300571 4631281

[ref21] Crowe A. Morgan E. H. (1992). Iron and transferrin uptake by brain and cerebrospinal fluid in the rat. Brain Res. 592, 8–16. doi: 10.1016/0006-8993(92)91652-U 1450923

[ref22] Cui Y. Zhang Y. Zhao X. Shao L. Liu G. Sun C.. (2021). ACSL4 exacerbates ischemic stroke by promoting ferroptosis-induced brain injury and neuroinflammation. Brain Behav. Immun. 93, 312–321. doi: 10.1016/j.bbi.2021.01.003, PMID: 33444733

[ref23] Daher R. Manceau H. Karim Z. (2017). Iron metabolism and the role of the iron-regulating hormone hepcidin in health and disease. Presse Med. 46, e272–e278. doi: 10.1016/j.lpm.2017.10.006 29129410

[ref24] Daubner S. C. Le T. Wang S. (2011). Tyrosine hydroxylase and regulation of dopamine synthesis. Arch. Biochem. Biophys. 508, 1–12. doi: 10.1016/j.abb.2010.12.017, PMID: 21176768 PMC3065393

[ref25] Deng Y.-F. Xiang P. Jing-Yi D. Liang J.-F. Li X. (2023). Intrathecal liproxstatin-1 delivery inhibits ferroptosis and attenuates mechanical and thermal hypersensitivities in rats with complete Freund’s adjuvant-induced inflammatory pain. Neural Regen. Res. 18, 456–462. doi: 10.4103/1673-5374.346547 35900446 PMC9396519

[ref26] Drakesmith H. Nemeth E. Ganz T. (2015). Ironing out Ferroportin. Cell Metab. 22, 777–787. doi: 10.1016/j.cmet.2015.09.006, PMID: 26437604 PMC4635047

[ref27] Fara A. Mitrev Z. Rosalia R. A. Assas B. M. (2020). Cytokine storm and COVID-19: a chronicle of pro-inflammatory cytokines. Open Biol. 10:200160. doi: 10.1098/rsob.200160 32961074 PMC7536084

[ref28] Farahani M. Niknam Z. Mohammadi Amirabad L. Amiri-Dashatan N. Koushki M. Nemati M.. (2022). Molecular pathways involved in COVID-19 and potential pathway-based therapeutic targets. Biomed. Pharmacother. 145:112420. doi: 10.1016/j.biopha.2021.112420, PMID: 34801852 PMC8585639

[ref29] Finazzi D. Arosio P. (2014). Biology of ferritin in mammals: an update on iron storage, oxidative damage and neurodegeneration. Arch. Toxicol. 88, 1787–1802. doi: 10.1007/s00204-014-1329-0, PMID: 25119494

[ref30] Fine J. M. Kosyakovsky J. Baillargeon A. M. Tokarev J. V. Cooner J. M. Svitak A. L.. (2020). Intranasal deferoxamine can improve memory in healthy C57 mice, suggesting a partially non-disease-specific pathway of functional neurologic improvement. Brain Behav. 10:e01536. doi: 10.1002/brb3.1536 31960628 PMC7066355

[ref31] Fratta Pasini A. M. Stranieri C. Girelli D. Busti F. Cominacini L. (2021). Is Ferroptosis a key component of the process leading to multiorgan damage in COVID-19? Antioxidants 10:1677. doi: 10.3390/antiox10111677 34829548 PMC8615234

[ref32] Frazer D. M. Anderson G. J. (2014). The regulation of iron transport. Biofactors 40, 206–214. doi: 10.1002/biof.1148 24132807

[ref33] Gaiatto A. C. Macedo T. A. Bibo N. D. G. M. Raimundo J. R. S. da Costa Aguiar Alves B. Gascón T.. (2023). COVID-19 compromises iron homeostasis: transferrin as a target of investigation. J. Trace Elem. Med. Biol. 76:127109. doi: 10.1016/j.jtemb.2022.127109, PMID: 36509021 PMC9694355

[ref34] Ganz T. (2005). Hepcidin--a regulator of intestinal iron absorption and iron recycling by macrophages. Best Pract. Res. Clin. Haematol. 18, 171–182. doi: 10.1016/j.beha.2004.08.020, PMID: 15737883

[ref35] Ganz T. (2006). Hepcidin--a peptide hormone at the interface of innate immunity and iron metabolism. Curr. Top. Microbiol. Immunol. 306, 183–198. doi: 10.1007/3-540-29916-5_7 PMID: 16909922

[ref36] Ganz T. (2007). Molecular control of iron transport. J. Am. Soc. Nephrol. 18, 394–400. doi: 10.1681/ASN.2006070802 17229910

[ref37] Ganz T. (2018). Iron and infection. Int. J. Hematol. 107, 7–15. doi: 10.1007/s12185-017-2366-2 29147843

[ref38] Ganz T. Nemeth E. (2012). Hepcidin and iron homeostasis. Biochim. Biophys. Acta 1823, 1434–1443. doi: 10.1016/j.bbamcr.2012.01.014, PMID: 22306005 PMC4048856

[ref39] Gille G. Reichmann H. (2011). Iron-dependent functions of mitochondria--relation to neurodegeneration. J. Neural Transm. (Vienna) 118, 349–359. doi: 10.1007/s00702-010-0503-7 21161302

[ref40] Ginzburg Y. Z. (2019). Hepcidin-ferroportin axis in health and disease. Vitam. Horm. 110, 17–45. doi: 10.1016/bs.vh.2019.01.002, PMID: 30798811 PMC7730607

[ref41] Graham R. M. Chua A. C. Herbison C. E. Olynyk J. K. Trinder D. (2007). Liver iron transport. World J. Gastroenterol. 13, 4725–4736. doi: 10.3748/wjg.v13.i35.4725, PMID: 17729394 PMC4611194

[ref42] Habib H. M. Ibrahim S. Zaim A. Ibrahim W. H. (2021). The role of iron in the pathogenesis of COVID-19 and possible treatment with lactoferrin and other iron chelators. Biomed. Pharmacother. 136:111228. doi: 10.1016/j.biopha.2021.111228, PMID: 33454595 PMC7836924

[ref43] Hambright W. S. Fonseca R. S. Chen L. Na R. Ran Q. (2017). Ablation of ferroptosis regulator glutathione peroxidase 4 in forebrain neurons promotes cognitive impairment and neurodegeneration. Redox Biol. 12, 8–17. doi: 10.1016/j.redox.2017.01.021, PMID: 28212525 PMC5312549

[ref44] Hao L. Mi J. Song L. Guo Y. Li Y. Yin Y.. (2021). SLC40A1 mediates ferroptosis and cognitive dysfunction in type 1 diabetes. Neuroscience 463, 216–226. doi: 10.1016/j.neuroscience.2021.03.009, PMID: 33727075

[ref45] Harrison P. M. Arosio P. (1996). The ferritins: molecular properties, iron storage function and cellular regulation. Biochim. Biophys. Acta 1275, 161–203. doi: 10.1016/0005-2728(96)00022-9, PMID: 8695634

[ref46] Hintze K. J. Theil E. C. (2006). Cellular regulation and molecular interactions of the ferritins. Cell. Mol. Life Sci. 63, 591–600. doi: 10.1007/s00018-005-5285-y 16465450 PMC11136433

[ref47] Hortová-Kohoutková M. Skotáková M. Onyango I. G. Slezáková M. Panovský R. Opatřil L.. (2023). Hepcidin and ferritin levels as markers of immune cell activation during septic shock, severe COVID-19 and sterile inflammation. Front. Immunol. 14:1110540. doi: 10.3389/fimmu.2023.1110540, PMID: 36776891 PMC9911830

[ref48] Hosseinzadeh P. Lu Y. (2016). Design and fine-tuning redox potentials of metalloproteins involved in electron transfer in bioenergetics. Biochim. Biophys. Acta 1857, 557–581. doi: 10.1016/j.bbabio.2015.08.006, PMID: 26301482 PMC4761536

[ref49] Huebers H. A. Huebers E. Csiba E. Rummel W. Finch C. A. (1983). The significance of transferrin for intestinal iron absorption. Blood 61, 283–290. doi: 10.1182/blood.V61.2.283.283 6821698

[ref50] Improta-Caria A. C. Soci Ú. P. R. Pinho C. S. Júnior R. A. De Sousa R. A. L. Bessa T. C. B. (2021). Physical exercise and immune system: perspectives on the COVID-19 pandemic. Rev. Assoc. Med. Bras. 67, 102–107. doi: 10.1590/1806-9282.67.suppl1.20200673, PMID: 34259761

[ref51] Júnior R. A. Durães A. Roever L. Macedo C. Aras M. G. Nascimento L.. (2021). The impact of COVID-19 on the cardiovascular system. Rev. Assoc. Med. Bras. 67, 163–167. doi: 10.1590/1806-9282.67.suppl1.20201063 34259776

[ref52] Kappert K. Jahic A. Tauber R. (2020). Assessment of serum ferritin as a biomarker in COVID-19: bystander or participant? Insights by comparison with other infectious and non-infectious diseases. Biomarkers 25, 616–625. doi: 10.1080/1354750X.2020.1797880 32700561

[ref53] Kaushal K. Kaur H. Sarma P. Bhattacharyya A. Sharma D. J. Prajapat M.. (2022). Serum ferritin as a predictive biomarker in COVID-19. A systematic review, meta-analysis and meta-regression analysis. J. Crit. Care 67, 172–181. doi: 10.1016/j.jcrc.2021.09.023, PMID: 34808527 PMC8604557

[ref54] Kempuraj D. Selvakumar G. P. Ahmed M. E. Raikwar S. P. Thangavel R. Khan A.. (2020). COVID-19, mast cells, cytokine storm, psychological stress, and neuroinflammation. Neuroscientist 26, 402–414. doi: 10.1177/1073858420941476, PMID: 32684080

[ref55] Kennedy M. C. Emptage M. H. Dreyer J. L. Beinert H. (1983). The role of iron in the activation-inactivation of aconitase. J. Biol. Chem. 258, 11098–11105. doi: 10.1016/S0021-9258(17)44390-0, PMID: 6309829

[ref56] Koorts A. M. Viljoen M. (2007). Ferritin and ferritin isoforms II: protection against uncontrolled cellular proliferation, oxidative damage and inflammatory processes. Arch. Physiol. Biochem. 113, 55–64. doi: 10.1080/13813450701422575, PMID: 17558604

[ref57] Kouhpayeh S. Shariati L. Boshtam M. Rahimmanesh I. Mirian M. Esmaeili Y.. (2021). The molecular basis of COVID-19 pathogenesis, conventional and nanomedicine therapy. Int. J. Mol. Sci. 22:5438. doi: 10.3390/ijms22115438, PMID: 34064039 PMC8196740

[ref58] Kumar P. Osahon O. Vides D. B. Hanania N. Minard C. G. Sekhar R. V. (2022). Severe glutathione deficiency, oxidative stress and oxidant damage in adults hospitalized with COVID-19: implications for GlyNAC (glycine and N-acetylcysteine) supplementation. Antioxidants 11:50. doi: 10.3390/antiox11010050 PMC8773164 35052554

[ref59] Lall M. M. Ferrell J. Nagar S. Fleisher L. N. McGahan M. C. (2008). Iron regulates L-cystine uptake and glutathione levels in lens epithelial and retinal pigment epithelial cells by its effect on cytosolic aconitase. Invest. Ophthalmol. Vis. Sci. 49, 310–319. doi: 10.1167/iovs.07-1041, PMID: 18172108

[ref60] Lee J. X. Chieng W. K. Abdul Jalal M. I. Tan C. E. Lau S. C. D. (2022). Role of serum ferritin in predicting outcomes of COVID-19 infection among sickle cell disease patients: a systematic review and meta-analysis. Front. Med. 9:919159. doi: 10.3389/fmed.2022.919159, PMID: 35712092 PMC9196080

[ref61] Lee K. E. Mo S. Lee H. S. Jeon M. Song J. S. Choi H. J.. (2021). Deferoxamine reduces inflammation and osteoclastogenesis in avulsed teeth. Int. J. Mol. Sci. 22:8225. doi: 10.3390/ijms22158225 34360988 PMC8348439

[ref62] Li Z. Liu T. Yang N. Han D. Mi X. Li Y.. (2020). Neurological manifestations of patients with COVID-19: potential routes of SARS-CoV-2 neuroinvasion from the periphery to the brain. Front. Med. 14, 533–541. doi: 10.1007/s11684-020-0786-5, PMID: 32367431 PMC7197033

[ref63] Li Y. Sun M. Fuyang Cao Y. Chen L. Z. Li H. Cao J.. (2022). The ferroptosis inhibitor liproxstatin-1 ameliorates LPS-induced cognitive impairment in mice. Nutrients 14:4599. doi: 10.3390/nu14214599, PMID: 36364859 PMC9656387

[ref64] Li S. Zhou C. Zhu Y. Chao Z. Sheng Z. Zhang Y.. (2021). Ferrostatin-1 alleviates angiotensin II (Ang II)- induced inflammation and ferroptosis in astrocytes. Int. Immunopharmacol. 90:107179. doi: 10.1016/j.intimp.2020.107179, PMID: 33278745

[ref65] Lino K. Guimarães G. M. C. Alves L. S. Oliveira A. C. Faustino R. Fernandes C. S.. (2021). Serum ferritin at admission in hospitalized COVID-19 patients as a predictor of mortality. Braz. J. Infect. Dis. 25:101569. doi: 10.1016/j.bjid.2021.101569, PMID: 33736948 PMC7959266

[ref66] Liu P. Feng Y. Li H. Chen X. Wang G. Xu S.. (2020). Ferrostatin-1 alleviates lipopolysaccharide-induced acute lung injury via inhibiting ferroptosis. Cell. Mol. Biol. Lett. 25:10. doi: 10.1186/s11658-020-00205-0, PMID: 32161620 PMC7045739

[ref67] Liu X. Yue D. Liu J. Cheng L. He W. Zhang W. (2023). Ferrostatin-1 alleviates cerebral ischemia/reperfusion injury through activation of the AKT/GSK3β signaling pathway. Brain Res. Bull. 193, 146–157. doi: 10.1016/j.brainresbull.2022.12.009, PMID: 36596364

[ref68] Liu W. Zhang S. Nekhai S. Liu S. (2020). Depriving Iron supply to the virus represents a promising adjuvant therapeutic against viral survival. Curr. Clin. Microbiol. Rep. 7, 13–19. doi: 10.1007/s40588-020-00140-w, PMID: 32318324 PMC7169647

[ref69] Matsui M. (1996). Early embryonic lethality caused by targeted disruption of the mouse thioredoxin gene. Dev. Biol. 178, 179–185. doi: 10.1006/dbio.1996.0208, PMID: 8812119

[ref70] McGahan M. C. Harned J. Mukunnemkeril M. Goralska M. Fleisher L. Ferrell J. B. (2005). Iron alters glutamate secretion by regulating cytosolic aconitase activity. Am. J. Physiol. Cell Physiol. 288, C1117–C1124. doi: 10.1152/ajpcell.00444.2004 15613494

[ref71] Miotto G. Rossetto M. Di Paolo M. L. Orian L. Venerando R. Roveri A.. (2020). Insight into the mechanism of ferroptosis inhibition by ferrostatin-1. Redox Biol. 28:101328. doi: 10.1016/j.redox.2019.101328, PMID: 31574461 PMC6812032

[ref72] Moos T. (2002). Brain iron homeostasis. Dan. Med. Bull. 49, 279–301. PMID: 12553165

[ref73] Morgan E. H. Oates P. S. (2002). Mechanisms and regulation of intestinal iron absorption. Blood Cells Mol. Dis. 29, 384–399. doi: 10.1006/bcmd.2002.0578 12547229

[ref74] Muhammad Y. Kani Y. A. Iliya S. Muhammad J. B. Binji A. Ahmad A. E.-F.. (2021). Deficiency of antioxidants and increased oxidative stress in COVID-19 patients: a cross-sectional comparative study in Jigawa, Northwestern Nigeria. SAGE Open Med. 9:205031212199124. doi: 10.1177/2050312121991246 PMC7871282 33614035

[ref75] Naderi S. Khodagholi F. Pourbadie H. G. Naderi N. Rafiei S. Janahmadi M.. (2023). Role of amyloid beta (25− 35) neurotoxicity in the ferroptosis and necroptosis as modalities of regulated cell death in Alzheimer’s disease. Neurotoxicology 94, 71–86. doi: 10.1016/j.neuro.2022.11.003, PMID: 36347329

[ref76] Nemeth E. Tuttle M. S. Powelson J. Vaughn M. B. Donovan A. Ward D. M.. (2004). Hepcidin regulates cellular iron efflux by binding to ferroportin and inducing its internalization. Science 306, 2090–2093. doi: 10.1126/science.1104742 15514116

[ref77] Neyens E. Baeyens J. (2003). A review of classic Fenton’s peroxidation as an advanced oxidation technique. J. Hazard. Mater. 98, 33–50. doi: 10.1016/S0304-3894(02)00282-0, PMID: 12628776

[ref78] Pandey K. Thurman M. Johnson S. D. Acharya A. Johnston M. Klug E. A.. (2021). Mental health issues during and after COVID-19 vaccine era. Brain Res. Bull. 176, 161–173. doi: 10.1016/j.brainresbull.2021.08.012, PMID: 34487856 PMC8414813

[ref79] Para O. Caruso L. Pestelli G. Tangianu F. Carrara D. Maddaluni L.. (2022). Ferritin as prognostic marker in COVID-19: the FerVid study. Postgrad. Med. 134, 58–63. doi: 10.1080/00325481.2021.1990091, PMID: 34613875 PMC8544665

[ref80] Paul B. T. Manz D. H. Torti F. M. Torti S. V. (2017). Mitochondria and Iron: current questions. Expert. Rev. Hematol. 10, 65–79. doi: 10.1080/17474086.2016.1268047, PMID: 27911100 PMC5538026

[ref81] Ponka P. Beaumont C. Richardson D. R. (1998). Function and regulation of transferrin and ferritin. Semin. Hematol. 35, 35–54. PMID: 9460808

[ref82] Ramsey A. J. Hillas P. J. Fitzpatrick P. F. (1996). Characterization of the active site iron in tyrosine hydroxylase. Redox states of the iron. J. Biol. Chem. 271, 24395–24400. PMID: 8798695 10.1074/jbc.271.40.24395

[ref83] Ren J. X. Sun X. Yan X. L. Guo Z. N. Yang Y. (2020). Ferroptosis in neurological diseases. Front. Cell. Neurosci. 14:218. doi: 10.3389/fncel.2020.00218, PMID: 32754017 PMC7370841

[ref84] Robbins A. H. Stout C. D. (1989). The structure of aconitase. Proteins 5, 289–312. doi: 10.1002/prot.340050406 2798408

[ref85] Salari N. Hosseinian-Far A. Jalali R. Vaisi-Raygani A. Rasoulpoor S. Mohammadi M.. (2020). Prevalence of stress, anxiety, depression among the general population during the COVID-19 pandemic: a systematic review and meta-analysis. Glob. Health 16, 1–11. doi: 10.1186/s12992-020-00589-w PMC7338126 32631403

[ref86] Savla S. R. Prabhavalkar K. S. Bhatt L. K. (2021). Cytokine storm associated coagulation complications in COVID-19 patients: pathogenesis and management. Expert Rev. Anti-Infect. Ther. 19, 1397–1413. doi: 10.1080/14787210.2021.1915129, PMID: 33832398 PMC8074652

[ref87] Seiler A. Schneider M. Forster H. Roth S. Wirth E. K. Culmsee C.. (2008). Glutathione peroxidase 4 senses and translates oxidative stress into 12/15-lipoxygenase dependent- and AIF-mediated cell death. Cell Metab. 8, 237–248. doi: 10.1016/j.cmet.2008.07.005, PMID: 18762024

[ref88] Sherry B. D. Fürstner A. (2008). The promise and challenge of Iron-catalyzed cross coupling. Acc. Chem. Res. 41, 1500–1511. doi: 10.1021/ar800039x 18588321

[ref89] Sousa D. Leoni R. A. Improta-caria A. C. Aras-júnior R. De Oliveira E. M. Soci Ú. P. R.. (2021). Physical exercise effects on the brain during COVID-19 pandemic: links between mental and cardiovascular health. Neurol. Sci. 42, 1325–1334. doi: 10.1007/s10072-021-05082-9, PMID: 33492565 PMC7829117

[ref90] Suriawinata E. Mehta K. J. (2022). Iron and iron-related proteins in COVID-19. Clin. Exp. Med. 1591–9528. doi: 10.1007/s10238-022-00851-y, PMID: 35849261 PMC9289930

[ref91] Tamir H. Liu K.-P. (1982). On the nature of the interaction between serotonin and serotonin binding protein: effect of nucleotides, ions, and sulfhydryl reagents. J. Neurochem. 38, 135–141. doi: 10.1111/j.1471-4159.1982.tb10864.x 7108523

[ref92] Taquet M. Sillett R. Zhu L. Mendel J. Camplisson I. Dercon Q.. (2022). Neurological and psychiatric risk trajectories after SARS-CoV-2 infection: an analysis of 2-year retrospective cohort studies including 1 284 437 patients. Lancet Psychiatry 9, 815–827. doi: 10.1016/S2215-0366(22)00260-7, PMID: 35987197 PMC9385200

[ref93] Taylor E. M. Morgan E. H. (1990). Developmental changes in transferrin and iron uptake by the brain in the rat. Brain Res. Dev. Brain Res. 55, 35–42. doi: 10.1016/0165-3806(90)90103-6, PMID: 2208639

[ref94] Todorich B. Pasquini J. M. Garcia C. I. Paez P. M. Connor J. R. (2009). Oligodendrocytes and myelination: the role of iron. Glia 57, 467–478. doi: 10.1002/glia.20784 18837051

[ref95] Wang L. Davis P. B. Volkow N. D. Berger N. A. Kaelber D. C. Xu R. (2022). Association of COVID-19 with new-onset Alzheimer’s disease. J. Alzheimers Dis. 89, 411–414. doi: 10.3233/JAD-220717, PMID: 35912749 PMC10361652

[ref96] Winterbourn C. C. (1995). Toxicity of iron and hydrogen peroxide: the Fenton reaction. Toxicol. Lett. 82-83, 969–974. doi: 10.1016/0378-4274(95)03532-X 8597169

[ref97] Xie Z. Wang X. Luo X. Yan J. Zhang J. Sun R.. (2023). Activated AMPK mitigates diabetes-related cognitive dysfunction by inhibiting hippocampal ferroptosis. Biochem. Pharmacol. 207:115374. doi: 10.1016/j.bcp.2022.115374, PMID: 36502872

[ref98] Xue H. Chen D. Zhong Y. K. Zhou Z. D. Fang S. X. Li M. Y.. (2016). Deferoxamine ameliorates hepatosteatosis via several mechanisms in Ob/Ob mice. Ann. N. Y. Acad. Sci. 1375, 52–65. doi: 10.1111/nyas.13174, PMID: 27447538

[ref99] Yang M. Lai C. L. (2020). SARS-CoV-2 infection: can ferroptosis be a potential treatment target for multiple organ involvement? Cell Death Discovery 6:130. doi: 10.1038/s41420-020-00369-w, PMID: 33251029 PMC7687212

[ref100] Yang W. S. Stockwell B. R. (2016). Ferroptosis: death by lipid peroxidation. Trends Cell Biol. 26, 165–176. doi: 10.1016/j.tcb.2015.10.014, PMID: 26653790 PMC4764384

[ref101] Yant L. J. Ran Q. Rao L. Van Remmen H. Shibatani T. Belter J. G.. (2003). The selenoprotein GPX4 is essential for mouse development and protects from radiation and oxidative damage insults. Free Radic. Biol. Med. 34, 496–502. doi: 10.1016/S0891-5849(02)01360-6, PMID: 12566075

[ref102] Ye Q. Zeng C. Luo C. Yuan W. (2020). Ferrostatin-1 mitigates cognitive impairment of epileptic rats by inhibiting P38 MAPK activation. Epilepsy Behav. 103:106670. doi: 10.1016/j.yebeh.2019.106670, PMID: 31864943

[ref103] Zandman-Goddard G. Shoenfeld Y. (2007). Ferritin in autoimmune diseases. Autoimmun. Rev. 6, 457–463. doi: 10.1016/j.autrev.2007.01.016 17643933

[ref104] Zhang R. Sun C. Chen X. Han Y. Zang W. Jiang C.. (2022). COVID-19-related brain injury: the potential role of ferroptosis. J. Inflamm. Res. 15, 2181–2198. doi: 10.2147/JIR.S353467, PMID: 35411172 PMC8994634

[ref105] Zhao Y. Luo Y. Liu Z. Chen Y. Wei L. Luo X.. (2023). Ferrostatin-1 ameliorates bupivacaine-induced spinal neurotoxicity in rats by inhibiting ferroptosis. Neurosci. Lett. 809:137308. doi: 10.1016/j.neulet.2023.137308 37244447

[ref106] Zhou C. Chen Y. Ji Y. He X. Xue D. (2020). Increased serum levels of hepcidin and ferritin are associated with severity of COVID-19. Med. Sci. Monit. 26:e926178. doi: 10.12659/MSM.926178 32978363 PMC7526336

[ref107] Zubair A. S. McAlpine L. S. Gardin T. Farhadian S. Kuruvilla D. E. Spudich S. (2020). Neuropathogenesis and neurologic manifestations of the coronaviruses in the age of coronavirus disease 2019: a review. JAMA 77, 1018–1010. doi: 10.1001/jamaneurol.2020.2065 PMC7484225 32469387

